# Brain and intersubjectivity: a Hegelian hypothesis on the self-other neurodynamics

**DOI:** 10.3389/fnhum.2014.00011

**Published:** 2014-01-24

**Authors:** Igor Marchetti, Ernst H. W. Koster

**Affiliations:** Department of Experimental-Clinical and Health Psychology, Ghent UniversityGhent, Belgium

**Keywords:** intersubjectivity, self-consciousness, default mode network, mirror neuron system, self concept, dehumanization, Hegel

## Introduction

Human beings live in a social context, where interacting with others is crucial for survival, and having a clear representation of both the “self” and the “other” is needed (Baumeister, 2011). Interestingly, neuroscience has traditionally adopted a Cartesian perspective by which the self is a solipsistic and self-sufficient unit, fundamentally unrelated to the other individuals' representation. Since the last decade, however, neuroscience is increasingly promoting research about how the “self” and the “other” are processed and represented at both intra- (Decety and Sommerville, 2003; Denny et al., 2012; Wagner et al., 2012; Molnar-Szakacs and Uddin, 2013) and inter-brain level (Dumas et al., 2010, 2012). Nevertheless, the specific way whereby neural self-other representations co-occur and exert influence on each other in order to promote higher-order functions necessary for social functioning remains largely unclear. We propose that the philosophical theory of *intersubjectivity* (Hegel, 1807/1977) could integrate neuroscience findings and, in turn, shed new light on the self-other dynamics at neurocognitive level.

## Hegel and self-other dynamics

Philosophy has intensively studied the interaction between the self and other, terming sometimes their interplay as *intersubjectivity*. In 1807, the German philosopher, Georg Wilhelm Friedrich Hegel, published the *Phenomenology of Spirit* (Hegel, 1807/1977), where he thoroughly investigated the progressive steps that from a relatively simple form of thinking, such as *consciousness*, lead to higher-order functions, such as *self-consciousness*.

According to Hegel, *consciousness* is the mental function that accounts for ordered experience and which provides us with a unified experience of reality, instead of a chaotic flow of meaningless information. By means of consciousness, we are therefore able to represent and process in a meaningful way the stimuli that fall in our phenomenal space. Recent theoretical efforts have associated this crucial mental function with specific brain dynamics: rest-stimulus interaction at the level of cortical and subcortical midline regions (Northoff, 2012).

Compared with earlier philosophers, Hegel took the notion of consciousness one step further. In line with his idealistic assumptions, he proposes that the mind actively “constructs” the objects of our knowledge, with no need for the latter to rely on cognitively inaccessible external entities. Earlier thinkers, such as Immanuel Kant, had indeed argued that these founding external entities were not comprehensible to our mind (i.e., thing-in-itself or *noumenon*) (Kain, 2005). Although his theory probably qualifies Hegel as one of the first radical constructivists, it also raises a fundamental question. If neither based on external reality nor on itself, on what is our self-consciousness based? Hegel (1807/1977) clearly answers that “*Self-consciousness exists in and for itself when, and by the fact that, it so exists for another; that is, it exists only in being acknowledged*” (paragraph 178) (Hegel, 1807/1977). Three elements are noteworthy here. First, Hegel proposes a condition of necessity in the self-other dyad, with each pole needing to be *recognized* by the other one (i.e., *mutual recognition* or *intersubjectivity*). Second, both the self and the other maintain their specific identity without merging into an undifferentiated matrix (Coelho and Figueiredo, 2003), in that the other is recognized as a separate subjectivity with whom a shared subjective state is possible (i.e., *recognition of the other*). Third, the self, at least in part, exists insofar as being constructed by the other that *recognizes* it back (i.e., *recognition of the self*). Without the other, the self lacks a fundamental reference to contrast itself with, and, in turn, it is bound not to emerge in the biological and psychological context (Baumeister, 2011). Importantly, a simple object fundamentally lacks self-consciousness and as such it is not eligible to recognize one's self as this would lead the latter to become “*a motionless tautology of I am I*” (paragraph 167) (Hegel, 1807/1977). In other words, if the other's recognition is lacking, I can experience events and objects (i.e., consciousness), but I cannot experience myself as a self-conscious agent (i.e., self-consciousness).

From the intersubjective perspective, the “other” is necessary. Not only must the other be physically present with its own body (Mead, 1934/2009; Russon, 1997), but the other must also recognize the subject as an intentional and self-conscious self (Kain, 2005). In order to process such fundamental but complex input, the brain is expected to be properly equipped to detect recognition by others.

## Intersubjectivity, default mode network, and mirror neuron system

How may Hegelian intersubjectivity inform our knowledge of the brain and, in turn, account for our social functioning? Intersubjectivity could shed light on recent neuroimaging findings by integrating two of the most active research lines in neuroscience, the Mirror Neurons System (MNS) and the Default Mode Network (DMN).

In the social context, the perception of a meaningful and goal-directed action leads to specific neural activation, regardless whether the action is performed by the subject itself or it is observed being performed by another actor (Iacoboni et al., 1999). The MNS has indeed been consistently associated with this function whereby the other's instrumental behavior is neuronally represented in the observer by means of a motor resonance mechanism. In other words, a significant gesture is encoded also in the observer's brain within its own motor schemata and this helps qualify the person of the observer as a (potentially) active and goal-directed actor. On the contrary, immobile objects or aimless actions do not elicit a similar response in the MNS (Preston and de Waal, 2002). Interestingly, already in the 1930's George Herbert Mead, a sociologist deeply influenced by Hegel's theory, stressed the importance of this “*conversation of gestures*” between individuals as the first step leading to higher levels of self-consciousness (Mead, 1934/2009; Markova, 1990). The MNS can therefore be considered as a neural tool that bridges the gap between the self and the other at the level of lower-order physical representation (Rizzolatti and Sinigaglia, 2010).

The DMN, a neural network highly active during rest, has been associated with psychological representing of others, that is mentalizing (Van Overwalle and Baetens, 2009; Schilbach et al., 2012). This cognitive ability to adopt someone else's perspective and internally represent mental states of others (i.e., beliefs, intentions, and goals) implies that we subsume the cognitive representation of other's mental activity into ours to some extent. Interestingly, while the MNS relies on the perception of other's embodied actions, mentalizing requires the ability to extract and understand goals and intentions of others. The latter implies that the other is recognized as an agent. Therefore, the DMN seems to subserve the mechanism through which the other psychologically resonates into one's mind as an intentional being (Molnar-Szakacs and Uddin, 2013).

As intersubjectivity is emerging as a promising perspective in neuroscience (Allen and Williams, 2011), we contend that it can meaningfully integrate these findings. Our Hegelian model suggests that it is the long-term interaction between the pre-reflective “*I*” (i.e., consciousness) and the other's representation, both at physical (Mead, 1934/2009; Russon, 1997) and psychological (Kain, 2005) level, that gives rise to a self-reflective “*I*” (i.e., self-consciousness). Hence, a self-conscious individual is capable not only of processing internal/external stimuli, but also of actively reflecting upon herself, which creates a network of information related to the self, such as attributes, beliefs, and traits (Baumeister, 2011).

In neurocognitive terms, we could say that self-awareness is the product of *interactions* between both lower- and higher-order functions, such as motor resonance and mentalizing subserved by the MNS and DMN, respectively. Crucially, this neural cooperation has been recently confirmed by a meta-analysis showing that self-specific activity emerges as interaction between the DMN (i.e., perigenual anterior cingulate cortex [pACC] and posterior cingulate cortex [PCC]), and MNS (i.e., left anterior insula [lAI] and right inferior frontal gyrus [rIFG]) (Qin and Northoff, 2011). Moreover, mentalizing about the self and other is associated with similar neurocircuitry, yet, the brain seems to be capable of distinguishing between them without equating one to the other (Lombardo et al., 2010).

In sum, Hegel's model of mutual recognition converges with neuroscience findings in that in order to understand ourselves we must rely on the same mechanisms that we use for understanding others. Self and other representations are strictly bond without one dissolving into the other (Coelho and Figueiredo, 2003; Lombardo et al., 2010). Notwithstanding this, the representation of others' minds and actions shapes our own mind, as both motor resonance mechanism (i.e., MNS) and mentalizing (i.e., DMN) are necessary to support and maintain the self-reflective self (Lombardo et al., 2010; Qin and Northoff, 2011; Sandrone, 2013). It is noteworthy that a similar hypothesis has been recently proposed by Timmermans et al. (2012). The authors propose that a set of neurobiological prediction-based mechanisms support our constant attempt to model other's mind and the related social interaction. Importantly, the very same mechanism is proposed to be crucial for developing self-consciousness too.

## The master-slave dynamic in the brain

Representation of the self and other is associated to a large extent with the same neural circuitry (Lombardo et al., 2010), and *mutual recognition* of self and other is required (Hegel, 1807/1977; Kain, 2005). However, this double bind implies a fragile equilibrium between the two components of the dyad. What if, for instance, the self fails to recognize the other? What if mutual recognition is deficient (Williams, 1997)?

Hegel (1807/1977) addresses this point by introducing the famous literary example of the master-slave dynamic, as follows. Given a couple of peers, one of the two could desire to undermine equilibrium by overpowering the other. Hence, one would become the “master” while the other becomes the“slave”. However, the situation is bound to turn out paradoxical. By not considering the other a full self-consciousness (i.e., objectification), the master prevents the slave from recognizing it back. Consequently, the master hinders the process of mutual recognition upon which it itself relies in order to emerge as a self-conscious individual. In cognitive terms, we could say that the individual enhances the threshold to receive the recognizing input that could structure it as self-conscious.

From this, several hypotheses derive. First, objectifying the other (i.e., negating its status of human being) leads, to a certain extent, also to a self-objectification, as the source of recognition is now lacking. Second, objectifying the other is expected to impact on the DMN and/or the MSN, the neurobiological systems maintaining self-consciousness. Preliminary findings support these hypotheses. First, being subjected to ostracisim (i.e., seeing the status of human being negated) leads the victims to judge both themselves and the ostracizer(s) less human (Bastian and Haslam, 2010). Second, actively engaging in ostracism makes the ostracizer feel less related to human beings (Legate et al., 2013). Third, a recent study showed that inducing participants to consider other human beings as objects leads to decreased activity of the DMN (Jack et al., 2013).

## Concluding remarks

Hegel's theory of intersubjectivity seems capable of shedding new light on the complex interaction between the self and the other at both neural and cognitive level. The interplay between the DMN and the MNS in supporting self-awareness may indeed be interpreted fruitfully by reintroducing the concept of *mutual recognition*.

Importantly, new hypotheses can be derived from the classic work of Hegel in order to better account for the interaction between DMN and MNS in both healthy and clinical samples. For instance, major psychopathologies characterized by abnormal self-other dynamic, such as schizophrenia, autism, and depression (Northoff, 2007; Crespi and Badcock, 2008; Mehta et al., 2012; Billeke et al., 2013; Gallese et al., 2013), represent key areas to test the explanatory power of the intersubjective theory. Finally, our neurophilosophical framework could be fruitfully integrated in a new branch of social neuroscience, namely the two-person neuroscience (Schilbach et al., 2013). By focusing on the neurocognitive basis of the interaction between two individuals, two-person neuroscience is developing new experimental paradigms and innovative methods to analyze real-time two-brain interplay, such as simultaneous neuroimaging recording or, so-called, hyperscanning (Dumas, 2011; Babiloni and Astolfi, in press). In sum, we argue that the integration of our Hegelian neurophilosophical approach and two-person neuroscience holds promise to convey innovative future perspectives in the field of social neuroscience.
